# Supplementary figures and images for: Characterisation of physicochemical parameters and antibacterial properties of New Caledonian honeys
Source: PLoS One. 2023 Oct 31;18(10):e0293730. doi: 10.1371/journal.pone.0293730 (PMC10617706; doi:10.1371/journal.pone.0293730)

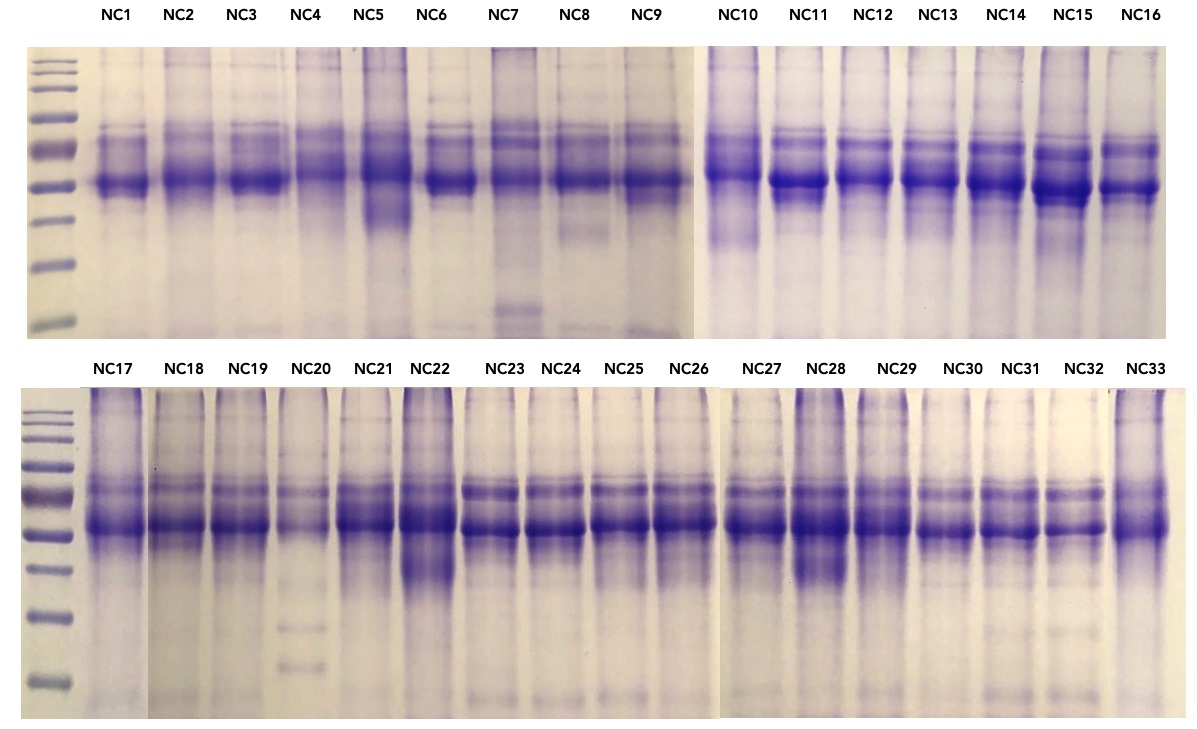

Supplement: S1 Fig — (TIF) [file pone.0293730.s001.tif]

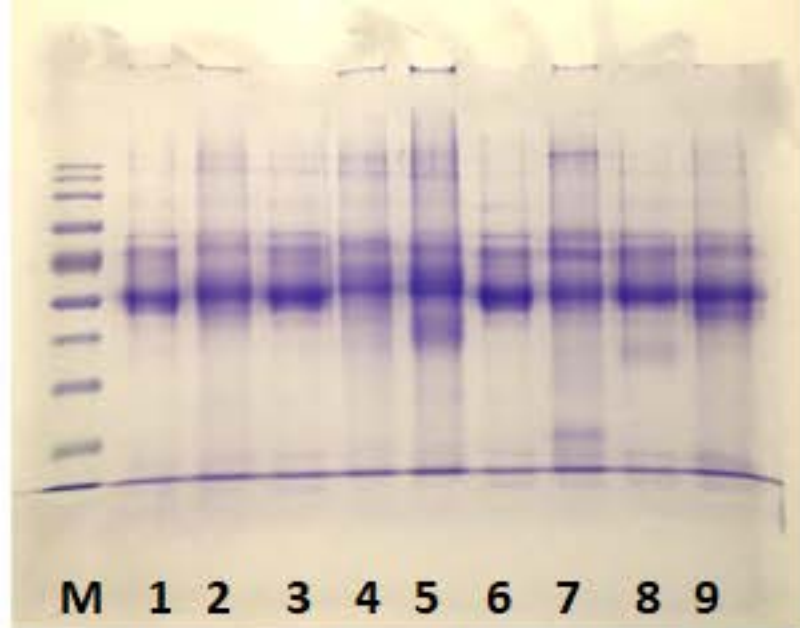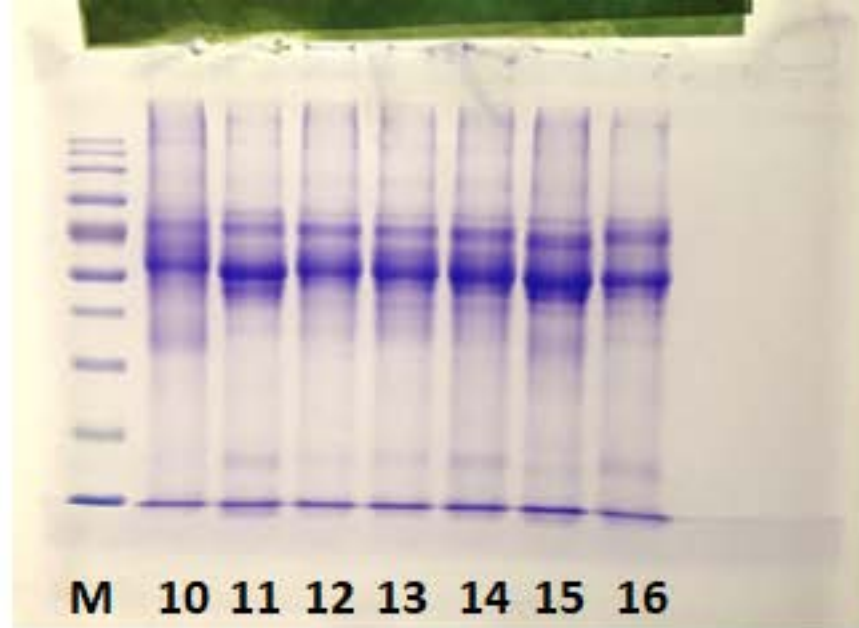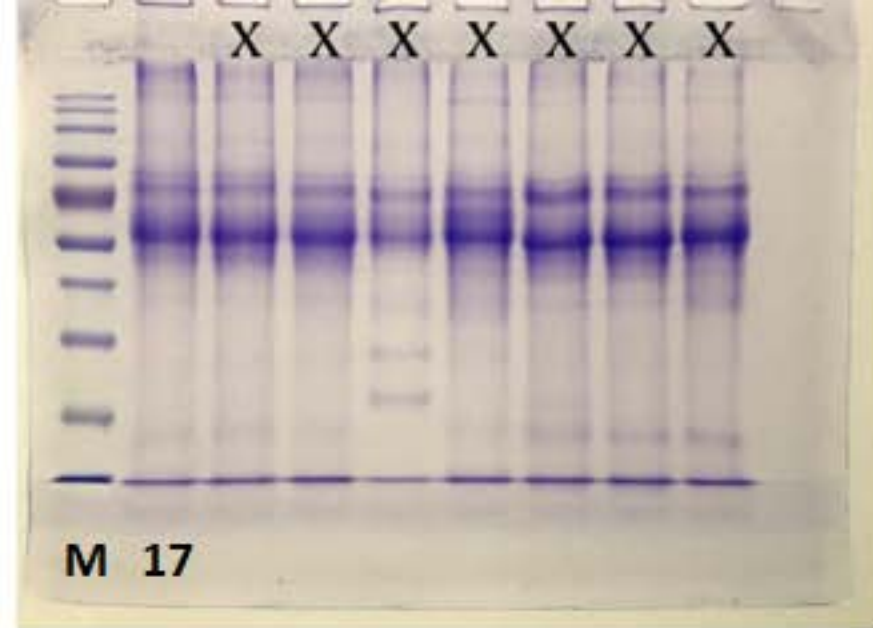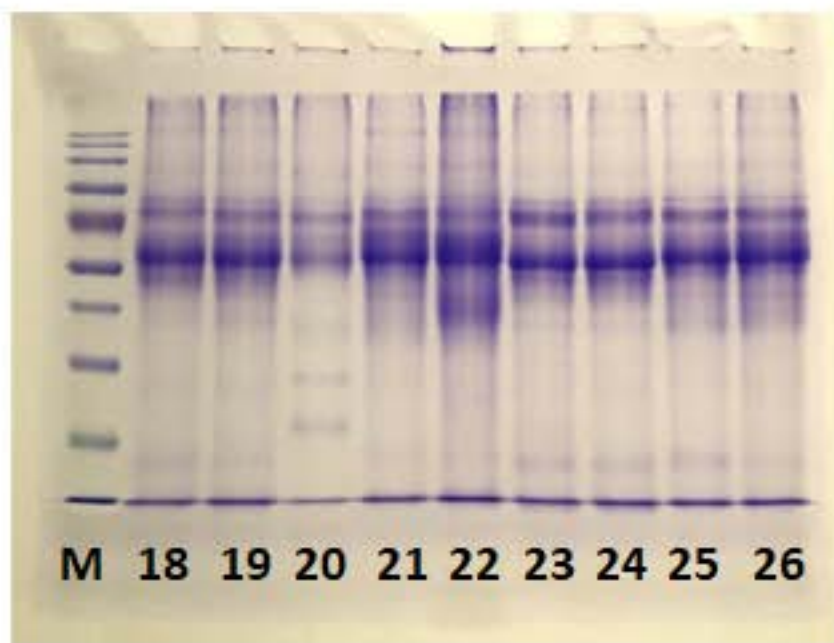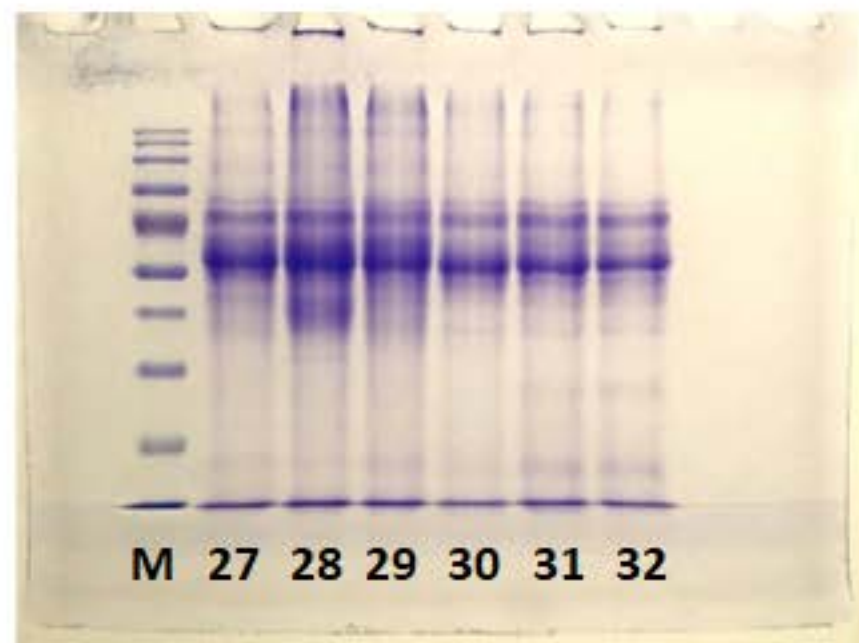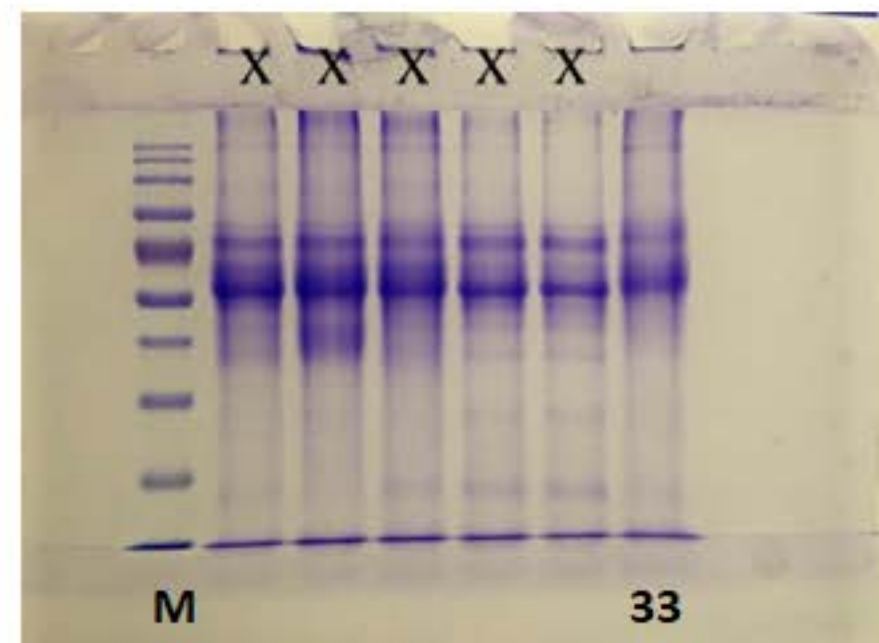

Supplement: S1 Raw images — (PDF) [file pone.0293730.s003.pdf]
