# Supplementary material for: Characterisation of physicochemical parameters and antibacterial properties of New Caledonian honeys
Source: PLoS One. 2023 Oct 31;18(10):e0293730. doi: 10.1371/journal.pone.0293730 (PMC10617706; doi:10.1371/journal.pone.0293730)
Supplement: S1 Table — (PDF) [file pone.0293730.s002.pdf]

Table S1. Qualitative pollen analysis of collected New Caledonian honey samples (n=33).

Sample No.	Pollen Type Classes		
	**≥46%**	**45–16%**	**15–4%**
**1**	*Myrtaceae*		
**2**		*Mimosaceae, Myrtaceae, Apocynaceae*	
**3**	*Myrtaceae*		
**4**		*Anacardiaceae, Cunoniaceae*	
**5**		*Fagaceae*	*Anacardiaceae*
**6**	*Myrtaceae*	*Apiaceae*	
**7**		*Myrtaceae, Casuarinaceae, Mimosaceae*	
**8**	*Myrtaceae*		*Cunoniaceae*
**9**	*Mimosaceae*	*Anacardiaceae*	
**10**	*Fagaceae*		
**11**		*Anacardiaceae, Myrtaceae*	*Mimosaceae*
**12**	*Anacardiaceae*	*Cunoniaceae*	
**13**	*Cunoniaceae*	*Anacardiaceae*	
**14**		*Anacardiaceae, Apiaceae, Cunoniaceae*	
**15**		*Salicaceae, Myrtaceae, Poaceae*	*Mimosaceae*
**16**		*Mimosaceae, Cunoniaceae, Myrtaceae*	
**17**		*Rhizophoraceae, Mimosaceae*	*Myrtaceae*
**18**	*Mimosaceae*		
**19**	*Anacardiaceae*	*Myrtaceae*	*Cunoniaceae*
**20**	*Cunoniaceae*		
**21**		*Cunoniaceae*	
**22**		*Cunoniaceae*	*Anacardiaceae*
**23**		*Anacardiaceae, Euphorbiaceae, Brassicaceae*	
**24**		*Anacardiaceae, Euphorbiaceae*	
**25**	*Anacardiaceae*		*Cunoniaceae*
**26**	*Anacardiaceae*	*Cunoniaceae*	
**27**	*Cunoniaceae*		
**28**		*Anacardiaceae, Apiaceae, Cunoniaceae*	*Salicaceae*
**29**	*Cunoniaceae*		
**30**	*Mimosaceae*	*Myrtaceae*	*Rosaceae*
**31**	*Apocynaceae*		*Zygophyllaceae, Anacardiaceae, Myrtaceae*
**32**	*Myrtaceae*	*Apocynaceae*	*Zygophyllaceae, Anacardiaceae*
**33**		*Myrtaceae, Mimosaceae*	*Cunoniaceae*
