# Supplementary material for: Characterisation of physicochemical parameters and antibacterial properties of New Caledonian honeys
Source: PLoS One. 2023 Oct 31;18(10):e0293730. doi: 10.1371/journal.pone.0293730 (PMC10617706; doi:10.1371/journal.pone.0293730)
Supplement: Attachment — Submitted filename: Reviosion 1.pdf [file pone.0293730.s004.pdf]

The manuscript entitled “Characterisation of physicochemical parameters and antibacterial properties of New Caledonian honeys” investigates the antibacterial activity of different honey samples in light of the specific hydrogen peroxide content. The manuscript seems well written, with the English requiring only minor adjustments (considering my knowledge). The topic is interesting and worthy of attention but as it stands the article has some shortcomings that do not allow it to be published.

In detail:

1. There is not a clear link between the introduction and the goals of the study.
2. The MMs are not clear on several points and need to be improved especially as regards the antimicrobial evaluations.
3. The results section shows data for which the methods of analysis have not been described (see subsection 3.1.).
4. The Results section reports information that looks more like discussion than results. This last is repeated several times so I would suggest a massive editing of the article to remove discussions from the result section.

Therefore, I suggest either rejecting the article or major revisions.

Abstract

In my opinion, the abstract does not elucidate well what the authors did during the study. The materials and methods section is not fully explanatory. The authors used abbreviations that may not be understood by a non-addicted reader. I suggest editing the abstract summarizing better all the sections of the study.

Introduction

The introduction is not badly written, but the authors should be focused better the aims of the study which are several and, in my opinion, are not well linked with the information reported in the introduction. I would suggest critically reviewing the introduction.

Line 51: Use another word instead of “consumption”.

Line 57: Improve the connection between this sentence and the periods before and after.

Lines 63-64: I would suggest moving this sentence before the previous period.

Line 70 and 72: Add references.

Line 82: add a reference.

Line 90: it is not clear what the authors mean by “uncover”. Please, improve.

Lines 90-91: It is not clear the connection between this sentence and the previous sentence. Could the author explain better, please?

Line 93: What do the authors mean by “high endemicity”? Please, improve and explain better.

Line 99: Why did the authors investigate the activity of glucose oxidase but they never discuss it in the introduction?

Line 101: What do the authors mean by "honey diluted? Please, improve.

Materials and methods

Lines 107-108: improved this sentence, it seems that the period is missing a final part.

Line 110: please, somewhere describe what UMF means.

Line 112: immediately before or after what?

Line 119: in my opinion, this paragraph does not make sense reported in this way. Improve scribing also how the samples were stored and prepared for the antibacterial activity. Furthermore, report the manufacturers where the bacteria were acquired.

Lines 120: isolates instead of isolate.

Line 125: what does "by laboratories" mean? Improve

Line 124: please, improve this paragraph by reporting at least a brief description of the methods used ("Harmonized methods of the internationals honey commission") and the manufacturers of the instruments used for the different parameters' determination.

Lines 159-161: it is unclear what and how was determined. The mic of what versus whom? diluted in broth how? Why? In what quantity? Improve.

Line 170: it is difficult for the reader to follow the article. The method is not well described. Why MHB "or" "diluted honey"? It seems that a part of the method is missing. Improve.

Line 172: visually in which way? Turbidity? Improve.

Lines 178-181: This section should be moved above before or inserted together with the description of the method adopted. It is not clear the difference between MIC and MBC.

Line 184: what do the authors mean by spotted?

Results

Line 206: This analysis was not defined in the MM. Add the methods used in the MM. Improve.

Line 225 legislative criterium of New Caledonia? Or another country/continent?

Lines 227-288: this looks more like discussions than results.

Lines 234-235: this looks more like discussions than results.

Please, rewrite the article so that only results and not discussions are reported in the results section. A it is, it is difficult to follow the results obtained by the analyses.

Discussion

The discussions seem well written and discretely discuss the results obtained. However, considering the need for major improvements in the results and the materials and methods sections, the discussions deserve further revision in the light of future updates by the authors.

Figure and tables

The captions of both figures and tables are fully explanatory and they are both well presented.
